# CommonMind Consortium provides transcriptomic and epigenomic data for Schizophrenia and Bipolar Disorder

**DOI:** 10.1038/s41597-019-0183-6

**Published:** 2019-09-24

**Authors:** Gabriel E. Hoffman, Jaroslav Bendl, Georgios Voloudakis, Kelsey S. Montgomery, Laura Sloofman, Ying-Chih Wang, Hardik R. Shah, Mads E. Hauberg, Jessica S. Johnson, Kiran Girdhar, Lingyun Song, John F. Fullard, Robin Kramer, Chang-Gyu Hahn, Raquel Gur, Stefano Marenco, Barbara K. Lipska, David A. Lewis, Vahram Haroutunian, Scott Hemby, Patrick Sullivan, Schahram Akbarian, Andrew Chess, Joseph D. Buxbaum, Greg E. Crawford, Enrico Domenici, Bernie Devlin, Solveig K. Sieberts, Mette A. Peters, Panos Roussos

**Affiliations:** 1 Pamela Sklar Division of Psychiatric Genomics, Department of Genetics and Genomic Sciences, Icahn School of Medicine at Mount Sinai, New York, New York USA; 2 Icahn Institute for Data Science and Genomic Technology, Department of Genetics and Genomic Sciences, Icahn School of Medicine at Mount Sinai, New York, New York USA; 3 Department of Psychiatry, Icahn School of Medicine at Mount Sinai, New York, New York USA; 4 Sage Bionetworks, Seattle, Washington USA; 5 Department of Pediatrics, Division of Medical Genetics, Duke University, Durham, North Carolina USA; 6 Human Brain Collection Core, National Institutes of Health, NIMH, Bethesda, Maryland USA; 7 Neuropsychiatric Signaling Program, Department of Psychiatry, Perelman School of Medicine, University of Pennsylvania, Philadelphia, Pennsylvania USA; 8 Neuropsychiatry Section, Department of Psychiatry, Perelman School of Medicine, University of Pennsylvania, Philadelphia, Pennsylvania USA; 9 Department of Psychiatry, University of Pittsburgh School of Medicine, Pittsburgh, Pennsylvania USA; 10 Department of Neuroscience, Icahn School of Medicine at Mount Sinai, New York, New York USA; 11 Psychiatry, JJ Peters VA Medical Center, Bronx, New York USA; 12 Department of Basic Pharmaceutical Sciences, Fred Wilson School of Pharmacy at High Point University, North Carolina, USA; 13 Department of Genetics, University of North Carolina at Chapel Hill, Chapel Hill, North Carolina USA; 14 Division of Psychiatric Epigenomics, Department of Psychiatry, Icahn School of Medicine at Mount Sinai, New York, New York USA; 15 Seaver Autism for Research and Treatment, Department of Psychiatry, Icahn School of Medicine at Mount Sinai, New York, New York USA; 16 Friedman Brain Institute, Icahn School of Medicine at Mount Sinai, New York, New York USA; 17 Laboratory of Neurogenomic Biomarkers, Department of Cellular, Computational and Integrative Biology (CIBIO), University of Trento, Trento, Italy

**Keywords:** Bipolar disorder, Bipolar disorder, Data integration, Schizophrenia, Schizophrenia

## Abstract

Schizophrenia and bipolar disorder are serious mental illnesses that affect more than 2% of adults. While large-scale genetics studies have identified genomic regions associated with disease risk, less is known about the molecular mechanisms by which risk alleles with small effects lead to schizophrenia and bipolar disorder. In order to fill this gap between genetics and disease phenotype, we have undertaken a multi-cohort genomics study of postmortem brains from controls, individuals with schizophrenia and bipolar disorder. Here we present a public resource of functional genomic data from the dorsolateral prefrontal cortex (DLPFC; Brodmann areas 9 and 46) of 986 individuals from 4 separate brain banks, including 353 diagnosed with schizophrenia and 120 with bipolar disorder. The genomic data include RNA-seq and SNP genotypes on 980 individuals, and ATAC-seq on 269 individuals, of which 264 are a subset of individuals with RNA-seq. We have performed extensive preprocessing and quality control on these data so that the research community can take advantage of this public resource available on the Synapse platform at http://CommonMind.org.

## Abstract (summary)

**Table:** 

Measurement(s)	genotype • assay for transposase-accessible chromatin using sequencing • transcription profiling assay • bipolar disorder • schizophrenia
Technology Type(s)	RNA sequencing • genotyping • ATAC-seq
Factor Type(s)	postmortem interval • age • biological sex • experimental group
Sample Characteristic - Organism	Homo sapiens

Machine-accessible metadata file describing the reported data: 10.6084/m9.figshare.9747479

## Background & Summary

Schizophrenia and bipolar disorder are serious mental illnesses (SMI) that affect more than 2% of adults^1,2^. There has been much recent progress in understanding the genetic basis for schizophrenia^3–8^. Yet understanding the molecular mechanisms linking risk loci to disease phenotypes, and characterizing disease pathways remains an open challenge. Here we have compiled a large-scale functional genomics resource in order to understand the interplay between genetic regulation, gene expression, chromatin accessibility and disease in the human brain. We have generated RNA-seq (n = 991) and ATAC-seq (n = 269) profiles from the dorsolateral prefrontal cortex specimens and SNP genotypes (n = 1076) from 4 separate brain banks. The genotype data includes 98 samples without RNA- or ATAC-seq data released with the anticipation of including functional data for these in a future release.

By providing an extensive public resource (http://CommonMind.org) of processed and quality controlled data, we aim to empower other researchers to apply novel methods and perform integrative analyses. Moreover, we provide raw and aligned data to enable custom reprocessing.

## Methods

### Multi-cohort data collection

This data release of the CommonMind Consortium^9^ is composed of 4 separate brain banks (Supplementary Fig. 1). Assembling individuals from multiple sites enables larger sample size; enables cross-site replication of findings; and reduces the effect of variables in sample collection, specimen preservation and generation of molecular data in any single site. The brain banks include 501 control individuals, 353 individuals with schizophrenia, as well as 132 individuals with either affective disorder, bipolar disorder or an undetermined disorder (Table 1). We note that age, percent male and postmortem interval varies substantially across cohorts.

**Table 1:** Multi-cohort demographics. Samples are control, undetermined (Other) or from individuals with schizophrenia (SCZ), affective disorders (AFF) or bipolar disorder (BP).

Diagnosis	Study	Brain Bank	N	Age (Years)	PMI	% Male
Control	CMC	MSSM	165	74.3 ± 17.5	10.8	51.5
		Penn	37	67.4 ± 15.5	13.4	48.6
		Pitt	83	48.0 ± 14.0	19	72.3
	CMC_HBCC	NIMH-HBCC	216	35.3 ± 20.4	29	71.3
SCZ	CMC	MSSM	147	72.8 ± 12.9	23.7	68.0
		Penn	57	79.2 ± 11.7	13.5	38.6
		Pitt	55	47.8 ± 13.1	20.2	76.4
	CMC_HBCC	NIMH-HBCC	94	49.9 ± 13.8	37	66.0
AFF/BP	CMC	MSSM	22	63.4 ± 16.0	15.7	32.8
		Penn	0			
		Pitt	35	45.5 ± 12.2	20.5	57.1
	CMC_HBCC	NIMH-HBCC	71	42.6 ± 14.4	29.8	67.6
Other	CMC_HBCC	NIMH-HBCC	4	31.5 ± 19.7	30.0	100.0

Mean age and mean postmortem interval (PMI) displayed (±s.d.).

#### MSSM - Mount Sinai NIH Brain Bank and Tissue Repository

Brain specimens are obtained from the Pilgrim Psychiatric Center, collaborating nursing homes, Veteran Affairs Medical Centers and the Suffolk County Medical Examiner’s Office. Disease diagnoses are made based on DSM-IV criteria and are obtained through direct assessment of subjects using structured interviews and/or through psychological autopsy by extensive review of medical records and informant or caregiver interviews. Consent is obtained from next of kin. The brain bank procedures are approved by the ISMMS IRB and exempted from further IRB review due to the collection and distribution of postmortem specimens.

#### Penn - University of Pennsylvania Brain Bank of Psychiatric illnesses and Alzheimer’s Disease Core Center

Brain specimens are obtained from the Penn Alzheimer’s Disease Core Center prospective collection. Disease diagnoses are made based on DSM-IV criteria and obtained through a clinical interview by psychiatrist and review of medical records. All procedures for Penn are approved by the Committee on Studies Involving Human Beings of the University of Pennsylvania, and the use of control postmortem tissues was considered exempted research in accordance with CFR 46.101 (b), item 65 of Federal regulations and University policy.

#### Pitt - University of Pittsburgh NIH NeuroBioBank Brain and Tissue Repository

Brain specimens are obtained during routine autopsies conducted at the Allegheny County Office of the Medical Examiner (Pittsburgh) following the consent of the next of kin. An independent committee of experienced research clinicians makes consensus DSM-IV diagnoses for all subjects on the basis of medical records and structured diagnostic interviews conducted with the decedent’s family members. All procedures for Pitt samples have been approved by the University of Pittsburgh’s Committee for the Oversight of Research involving the Dead and Institutional Review Board for Biomedical Research.

#### HBCC - NIMH Human Brain Collection Core

Brain specimens are obtained under protocols approved by the CNS IRB (NCT03092687), with the permission of the next-of-kin (NOK) through the Offices of the Chief Medical Examiners (MEOs) in the District of Columbia, Northern Virginia and Central Virginia. All specimens were characterized neuropathologically, clinically and toxicologically. A clinical diagnosis was obtained through family interviews and review of medical records by two psychiatrists based on DSM-IV criteria. Non-psychiatric controls were defined as having no history of a psychiatric condition or substance use disorder and negative toxicology measures.

### Study description

#### MSSM/Penn/Pitt cohorts (the “CMC” study)

This study includes data from the dorsolateral prefrontal cortex provided by the MSSM, Penn, and Pitt brain banks. Tissue for the study was dissected at each brain bank and shipped to the Icahn School of Medicine at Mount Sinai (ISMMS) for nucleotide isolation and data generation in one facility in order to reduce site-specific sources of technical variation. Postmortem tissue from schizophrenia and bipolar disorder cases were included if they met the diagnostic criteria in DSM-IV for schizophrenia or schizoaffective disorder, or for bipolar disorder, as determined in consensus conferences after review of medical records, direct clinical assessments, and interviews of care providers. Cases that had a history of Alzheimer’s disease, and/or Parkinson’s disease, or acute neurological insults (anoxia, strokes, and/or traumatic brain injury) immediately prior to death, or were on ventilators near the time of death, were excluded. Eight samples were classified as a mood disorder other than bipolar post sample collection and have been labeled as AFF in the clinical file.

#### HBCC cohort (the “CMC_HBCC” study)

This study includes data from the dorsolateral prefrontal cortex from the NIMH HBCC brain bank. Tissue for the study was dissected at the NIMH HBCC and shipped to the Icahn School of Medicine at Mount Sinai (ISMMS) for nucleotide isolation and data generation in one facility in order to reduce site-specific sources of technical variation. Tissue pH, RNA integrity number (RIN), and postmortem interval (PMI) were ascertained.

### Tissue dissection

#### MSSM/Penn/Pitt cohorts

Samples were dissected at each of the brain banks and shipped to Icahn School of Medicine - Mt Sinai (ISMMS) for sample preparation, genotyping, and RNA-sequencing. Dissection protocols were as follows:

- *MSSM*: All samples for the study were dissected from the left hemisphere of fresh frozen coronal slabs cut at autopsy from the dorsolateral prefrontal cortex from Brodmann areas 9 or 46. Immediately after dissection, samples were cooled to −190 °C and dry homogenized to a fine powder using a L-N2 cooled mortar and pestle. Tissue was transferred on dry ice to ISMMS as a dry powder for DNA and RNA extraction.
- *Penn*: At autopsy, right or left hemisphere of each brain was blocked into coronal slabs, which were immediately frozen and stored at −80 °C. For this study, the Brodmann areas 9 or 46 were dissected from either the left or right hemisphere and pulverized in liquid nitrogen. The tissue was shipped in tubes appropriate for DNA or RNA extraction to ISMMS as homogenized tissue in trizol for RNA extraction and dry pulverized tissue for DNA extraction.
- *Pitt*: At autopsy, the right hemisphere of each brain was blocked coronally, immediately frozen and stored at −80 °C. Samples for the study contained only the gray matter of DLPFC where Brodmann area 9 from the right hemisphere was cut on a cryostat and collected in tubes appropriate for DNA or RNA extraction. The DNA and RNA tubes were shipped on dry ice to ISMMS as homogenized tissue in trizol for RNA extraction and thinly sliced tissue for DNA extraction. Specimens from Pitt were provided as matched case/control pairs. These were perfectly matched for sex, and as closely as possible for age and race. Members of a pair were always processed together.

#### HBCC cohort

Samples for the study were dissected from either the left or right hemisphere of fresh frozen coronal slabs cut and frozen and stored at −80 °C. Brodmann areas 9 and 46 on the dorsolateral surface of the prefrontal cortex were dissected from the frozen slabs under the direction of a neuropathologist. The samples were shipped on dry ice to ISMMS as homogenized tissue in Trizol for RNA extraction.

### RNA preparation

#### MSSM/Penn/Pitt cohorts

Dorsolateral Prefrontal Cortex: Total RNA from 670 samples was isolated from approximately 50 mg homogenized tissue in Trizol using the RNeasy kit according to manufacturer’s protocol. Samples from all brain banks were processed together in batches of 12 and the Pitt matched case/control pairs were always processed in the same batch. 10 control samples were processed as part of both a schizophrenia and bipolar pair and therefore found as duplicate samples in the dataset. The order of extraction for schizophrenia-affected and control samples was assigned randomly with respect to brain bank, diagnosis and all other sample characteristics. Because the bipolar-affected and matched controls were not available until after the processing of the schizophrenia and controls was underway, these samples were randomized among the remaining 132 schizophrenia and control samples for extraction. The mean total RNA yield was 15.3 ug. The RNA Integrity Number (RIN) was determined by fractionating RNA samples on the 6000 Nano chip (Agilent Technologies) on the Agilent 4200 TapeStation. Samples with RIN > =5.5 were included in the sequencing set, resulting in 46 samples with RIN < 5.5 to be excluded from the study. An additional 18 samples were removed post sequencing due to sample genotype variant inconsistencies and low reads resulting in a final dataset of 606 samples.

#### HBCC cohort

Dorsolateral Prefrontal Cortex: Total RNA from 469 HBCC samples was isolated from approximately 100 mg pulverized tissue from each sample by Trizol/chloroform extraction and purification with the Qiagen RNeasy kit (Cat #74106) according to manufacturer’s protocol. Samples were processed in randomized batches of 12. The order of extraction was assigned randomly with respect to diagnosis and all other sample characteristics. The mean total RNA yield was 24.2 ug. The RNA Integrity Number (RIN) was determined by fractionating RNA samples on the 4200 Agilent TapeStation System. Samples with RIN > =5.5 were included in the sequencing set, resulting in 69 samples with RIN < 5.5 to be excluded from the study. An additional 11 samples were removed post sequencing due to sample genotype variant inconsistencies, and 4 duplicate samples were removed (the sample with the highest RIN was kept) resulting in a final dataset of 385 samples.

### RNA sequencing

RNA sequencing raw and gene counts is provided for 991 samples originating from 981 unique donors, where 10 ‘Pitt’ control samples were run in duplicate. Data was generated, QC’ed, processed and quantified as follows:

#### MSSM/Penn/Pitt cohorts

RNA library preparation and sequencing: Processing order was randomized prior to ribosomal RNA depletion, and samples were processed in batches of 8. In order to expedite sequencing, processing began before extraction was complete and randomization occurred among all available extracted samples in sets of 120 to 226. Briefly, rRNA was depleted from about 1 ug of total RNA using Ribozero Magnetic Gold kit (Illumina/Epicenter Cat #MRZG12324) to enrich polyadenylated coding RNA and non-coding RNA. The Pitt case/control pairs were batched together in each processing step, including Ribozero depletion, sequence library preparation and sequencing lane. The sequencing library was prepared using the TruSeq RNA Sample Preparation Kit v2 (RS-122-2001-48 reactions) in batches of 24 samples. The insert size and DNA concentration of the sequencing library was determined on Agilent Bioanalyzer and Qubit, respectively. A pool of 10 barcoded libraries were layered on a random selection of two of the eight lanes of the Illumina flow cell at appropriate concentration and bridge amplified to ~250 million raw clusters. One-hundred base pair paired end reads were obtained on a HiSeq 2500. The sequencing data generated was simultaneously transferred (in real time) to storage computer cluster and then transferred to high performance computer cluster. The sequence data was processed for primary analysis to generate QC values. Data is provided for those samples that passed all of the following QC filters: samples were required to have had a minimum of 25 million read pairs and less than 5% rRNA alignment. This gives a mean of 40.1 million, median of 39 million and maximum of 103.9 million read pairs.

#### HBCC cohort

RNA library preparation and sequencing: All samples submitted to the New York Genome Center for RNA-seq were prepared for sequencing in randomized batches of 94. The sequencing libraries were prepared using the KAPA Stranded RNA-seq Kit with RiboErase (KAPA Biosystems). rRNA was depleted from 1ug of RNA using the KAPA RiboErase protocol that is integrated into the KAPA Stranded RNA-seq Kit. The insert size and DNA concentration of the sequencing library was determined on Fragment Analyzer Automated CE System (Advanced Analytical) and Quant-iT PicoGreen (ThermoFisher) respectively. A pool of 10 barcoded libraries were layered on a random selection of two of the eight lanes of the Illumina flow cell at appropriate concentration and bridge amplified to ~250 million raw clusters. One-hundred base pair paired end reads were obtained on a HiSeq 2500. Data is provided for those samples that passed all of the following QC filters: samples were required to have had a minimum of 25 million read pairs and less than 5% rRNA alignment. This gives a mean of 56.5 million, median of 56.4 million and maximum of 74.4 million read pairs.

#### Uniform computational processing

Mapping and quantification of genes, exons and transcripts: The raw reads were trimmed with Trimmomatic (v0.36)^10^ and then mapped to human reference genome GRCh38.v24 (ftp://ftp.ebi.ac.uk/pub/databases/gencode/Gencode_human/release_24/GRCh38.primary_assembly.genome.fa.gz) using STAR (v2.5.3a)^11^. The BAM files that were generated contain the mapped paired-end reads, including those spanning splice junctions. Following read alignment, expression quantification was performed at the transcript isoform level using RSEM (v1.3.0)^12^ and then summarized at the gene level. Gene quantifications correspond to GENCODE v27 (ftp://ftp.ebi.ac.uk/pub/databases/gencode/Gencode_human/release_27/gencode.v27.annotation.gtf.gz). Quality control metrics were reported with RNA-SeqQC (v1.1.7)^13^. All analysis used log2 counts per million (CPM) following TMM normalization^14^ implemented in edgeR (v3.22.5)^15^. Correction for GC content bias was performed with cqn (v1.26.0)^16^. Genes with over 1 CPM in at least 50% of the experiments were retained.

### DNA Preparation

#### MSSM/Penn/Pitt cohorts

DNA was isolated from approximately 10 mg dry homogenized tissue coming from the same dissected samples as the RNA isolation. The thinly sliced tissue from Pitt was homogenized before DNA isolation. All DNA isolation was done using the Qiagen DNeasy Blood and Tissue Kit according to manufacturer’s protocol. DNA yield was quantified using Thermo Scientific’s NanoDrop. The mean yield was 12.6 ug (+/−4.6), the mean ratio of 260/280 was 2.0 (+/−0.1) and the mean ratio of 260/230 was 1.9 (+/−2.3).

#### HBCC cohort

Varying amounts of pulverized cerebellar tissue were used (45 to 80 mg) for DNA extraction. The QIAamp DNA mini Kit (Qiagen) method was used. The tissue was initially lysed using Tissue Lyser (Qiagen) and extractions proceeded according to manufacturer’s protocol. DNA was captured in 500uL elution buffer. The concentrations were measured using Thermo Scientific’s NanoDrop 1000/NanoDrop ONE. The mean yield was 128.85 uG (+/−79.48), the mean ratio of 260/280 was 1.87 (+/−0.105), and the mean ratio of 260/230 was 2.48 (+/−1.75).

### SNP array processing, imputation

For the MSSM/Penn/Pitt cohorts, genotyping was performed on the Illumina Infinium HumanOmniExpressExome 8 v 1.1b chip (Catalog #: WG-351-2301) using the manufacturer’s protocol as previously described^9^. The HBCC samples were genotyped on one of 3 different Illumina gene chips: HumanHap650Y, Human1M-Duo, and HumanOmni5M-Quad using the manufacturer’s protocol as follows: Approximately, 400 ng DNA was used and each DNA sample was QC tested for 260/280 ratio by nanodrop and DNA band intactness on 2% agarose gel. Briefly, the samples were whole-genome amplified, fragmented, precipitated and resuspended in appropriate hybridization buffer. Denatured samples were hybridized on prepared Illumina Quad Bead Chips. After hybridization, the Bead Chip oligonucleotides were extended by a single fluorescent labeled base, which was detected by fluorescence imaging with an Illumina Bead Array Reader, iScan.

Genotype calling, QC and imputation proceeded separately by gene chip set. Normalized bead intensity data obtained for each sample were called using Illumina Genome Studio with cluster position files provided by Illumina, and fluorescence intensities were converted into SNP genotypes separately for each gene chip. Initial QC was performed using PLINK^17^ to remove markers with: zero alternate alleles, genotyping call rate ≤ 0.98, Hardy-Weinberg *P* value < 5 × 10^−5^, and individuals with genotyping call rate < 0.90. Samples were then imputed to HRC (r1.1 2016)^18^, as follows: if necessary, marker positions were lifted-over to GRCh37 and aligned to the HRC loci using HRC-1000G-check-bim-v4.2 (https://www.well.ox.ac.uk/~wrayner/tools/), which checks the strand, alleles, position, reference/alternate allele assignments and frequencies of the markers, removing A/T & G/C single nucleotide polymorphisms (SNPs) with minor allele frequency (MAF) > 0.4, SNPs with differing alleles, SNPs with > 0.2 allele frequency difference between the genotyped samples and the HRC samples, and SNPs not in reference panel. Imputation was performed via the Michigan Imputation Server^19^ using Eagle v2.3^20^ as the phasing algorithm. Since all imputation was performed using the GRCh37, the genotype data was subsequently lifted over to GRCh38 for downstream integrating with functional genomics assays.

### ATAC-seq

#### Library preparation and sequencing

A total number of 314 frozen pulverized brain samples were received from the Mt. Sinai Brain Repository, and processed for ATAC-seq as described^21^. Briefly, 20 mg of tissue was pulverized in liquid nitrogen and thawed in 1 ml of nuclear isolation buffer (20 mM Tris-HCl, 50 mM EDTA, 5 mM spermidine, 0.15 mM spermine, 0.1% mercaptoethanol, 40% glycerol, pH 7.5). Samples were mixed by inversion, filtered from large pieces of tissue through Miracloth, centrifuged at 1100 × g for 10 min at 4 °C, pellets washed with 50 μl Reduced Swing buffer, centrifuged again, and supernatants were removed. The nuclear pellets were resuspended in Tn5 transposase reaction mix, barcoded, combined into pools, and used for sequencing^22^. Each pool contained eight randomly selected samples that were balanced by case–control status and gender. Then, each pool was sequenced at Duke Sequencing and Genomic Technologies shared resource on two lanes of Illumina HiSeq 2000 or HiSeq 4000 obtaining 2 × 125 or 2 × 151 single- or paired-end reads. Since only eight samples were sequenced in the single-end mode and all showed different epigenomics profile based on multidimensional scaling analysis, we decided to exclude them. After additional quality controls (see below), 269 ATAC-seq libraries were retained^21^.

#### Alignment of reads

Each set of pair-end reads was processed by Trimmomatic^10^ to remove low-quality base pairs and sequence adapters. Reads were subsequently aligned to the GRCh38 (http://hgdownload.cse.ucsc.edu/goldenPath/hg38/bigZips/analysisSet/hg38.analysisSet.chroms.tar.gz) analysis set reference genome with the pseudoautosomal region masked on chromosome Y with the STAR aligner^11^. This yielded for each sample a BAM file of mapped paired-end reads sorted by genomic coordinates. From these files, reads that mapped to multiple loci or to the mitochondrial genome were removed using samtools^23^ and duplicated reads were removed with PICARD (http://broadinstitute.github.io/picard).

#### Peak calling and annotation

To create a final peakset, we subsampled and merged BAM-files separately for schizophrenia and control samples. For each, a total of one billion paired-end reads were used, corresponding to 6.99 million read pairs sample per schizophrenia sample and 7.94 million read pairs per control sample. We called peaks separately on these two merged BAM files, and merging these two peaksets into a single consensus peakset. Finally, we removed peaks overlapping ENCODE blacklisted regions of low read mappability or repeated sequence (https://raw.githubusercontent.com/mills-lab/svelter/master/Support/GRCh38/Exclude.GRCh38.bed). For each peak, we assigned the closest gene and the genomic context of an ATAC-seq OCR using ChIPSeeker^24^; transcript database was built by GenomicFeatures^25^ upon ENSEMBL genes.

### Macaque RNA-seq (the “CMC_Macaque” study)

Subjects from a cohort of N = 34 Rhesus macaques born between 1995 and 2004 were randomly selected for four treatment groups: 7 for high doses of haloperidol (4 mg/kg/d), 10 low doses of haloperidol (0.14 mg/kg/d), 9 clozapine (5.2 mg/kg/d), and 8 vehicle (Table 2). Monkeys were administered the antipsychotic drugs orally for six months, mixed with powdered sugar and given in peanut butter or fruit treats. Monkeys were raised at Wake Forest University and received standard enrichment, including social enrichment, human interaction, variety in diet, and age-appropriate objects as dictated by the Animal Welfare Act and the Emory University and Wake Forest School of Medicine policies for nonhuman primate environmental enrichment. Animal care procedures strictly followed the National Institutes of Health Guide for the Care and Use of Laboratory Animals and were approved by the Institutional Animal Care and Use Committees of Emory University and Wake Forest School of Medicine. Monkeys were killed for analysis and necropsied on average at age 6.2 years (range between 3.6 and 8.2 years old) after the six-month treatment protocol by an overdose of barbiturate and transcardially perfused with ice cold saline. The brains were removed and cut into 4-mm slabs in the coronal plane using a brain matrix (EMS, Fort Washington, PA) and immediately frozen and stored at −80 °C.

**Table 2:** Summary of key variables from Rhesus macaques drug response experiments.

Treatment	Number Animals	Sex - F/M
Clozapine	9	5/4
Haloperidol-high	7	4/3
Haloperidol-low	10	5/5
Placebo	8	4/4

A file of treatment variables can be found on Synapse^26^.

#### Sample preparation

Tissue was dissected from slabs of the right hemisphere that included the basal ganglia from the rostral pole to the beginning of the anterior commissure. The DLPFC was dissected from the dorsal and ventral banks of the principal sulcus (Area 46) and pulverized. Samples were shipped to ISMMS for sample preparation and RNA-sequencing.

#### RNA isolation

Dorsolateral Prefrontal Cortex: Total RNA was isolated from approximately 100 mg homogenized tissue in Trizol using the RNeasy kit according to manufacturer’s protocol and processed together in treatment and sex randomized batches of 12 samples. The mean total RNA yield was 28.5 ug. The RNA Integrity Number (RIN) was determined by fractionating RNA samples on the 6000 Nano chip (Agilent Technologies) on the Agilent 2100 Bioanalyzer. Samples with RIN > =5.5 were included in the sequencing set, resulting in 2 samples with RIN < 5.5 being excluded from the study. Among the remaining samples the mean RIN was 7.3 and the mean ratio of 260/280 was 2.1.

#### Sequencing

Raw and quantified RNA-seq expression data is provided for 32 samples consisting of data from 32 unique animals. Data was generated, QC’ed, processed and quantified as follows:

Processing order was randomized prior to ribosomal RNA depletion, and samples were processed in batches of 9 or 8. rRNA was depleted from about 1 ug of total RNA using Ribozero Magnetic Gold kit (Illumina/Epicenter Cat #MRZG12324) to enrich polyadenylated coding RNA and non-coding RNA. The sequencing library was prepared using the TruSeq RNA Sample Preparation Kit v2 (RS-122-2001-48 reactions) in batches of 17 samples. The insert size and DNA concentration of the sequencing library was determined on Agilent Bioanalyzer and Qubit, respectively. A pool of 8 or 9 barcoded libraries were layered on a random selection of two of the eight lanes of the Illumina flow cell at appropriate concentration and bridge amplified to ~250 million raw clusters. One-hundred base pair paired end reads were obtained on a HiSeq 2500. The sequencing data were simultaneously transferred (in real time) to storage computer cluster and then transferred to high performance computer cluster. The sequence data was processed for primary analysis to generate QC values. All data provided passed all of the following QC filters: samples were required to have a minimum of 50 million total reads and less than 5% rRNA alignment.

#### Mapping and quantification of genes

Reads were mapped to the macaque reference genome and transcriptome (mmul1) using STAR^11^ and featureCounts^27^.

## Data Records

RNA-seq, ATAC-seq, SNP genotypes, and sample metadata is available from the CommonMind Consortium Knowledge Portal using the Synapse platform at http://CommonMind.org. This includes a complete table of files described in this manuscript is available on Synapse. Data is either open, where the only requirement is to acknowledge data contributors in publications, or controlled. Controlled data access application must be placed through the NIMH Repository and Genomics Resources (https://www.nimhgenetics.org/resources/commonmind). The CommonMind Consortium resource includes processed data as well as raw FASTQ and BAM files. A tutorial on how to access the data using the Synapse platform is available at https://docs.synapse.org/articles/downloading_data.html.

A complete list of files available through the consortium, including information about access type (open or controlled), can be found on Synapse at http://CommonMind.org:

Important files for this resource include:

1. Sample metadata files:
  - ATAC-seq^28^
  - RNA-seq^29^
  - Genotypes^30^
  - Clinical^31^
2. Genotypes QCd - CMC study^32^
3. Genotypes QCd - CMC_HBCC study^33^
4. Genotypes Imputed - CMC/CMC_HBCC^34^
5. RNA-seq expression quantifications^35^
6. RNA-seq BAM files^36^
7. RNA-seq FASTQ files with remaining unmapped reads^37^
8. ATAC-seq read count matrix^38^
9. ATAC-seq fastq^39^
10. ATAC-seq consensus BigWig^40^
11. ATAC-seq BigWig per sample^41^
12. ATAC-seq consensus peaks^42^
13. ATAC-seq peaks per sample^43^
14. ATAC-seq BAM files^44^
15. Rhesus macaques metadata^26^
16. Rhesus macaque RNA-seq expression quantifications^45^
17. Rhesus macaque RNA-seq BAM files^46^
18. Rhesus macaque RNA-seq files with remaining unmapped reads^47^

We note that this data release supersedes the initial data release from Fromer, *et al*.^9^. The original analysis results are available on from this Synapse project.

## Technical Validation

We note that qPCR validation of gene expression levels of 13 genes in 57 schizophrenia and 57 matched control individuals from the University of Pittsburgh brain bank was performed in the original paper^9^.

### RNA-seq quality control

**Fig. 1:**
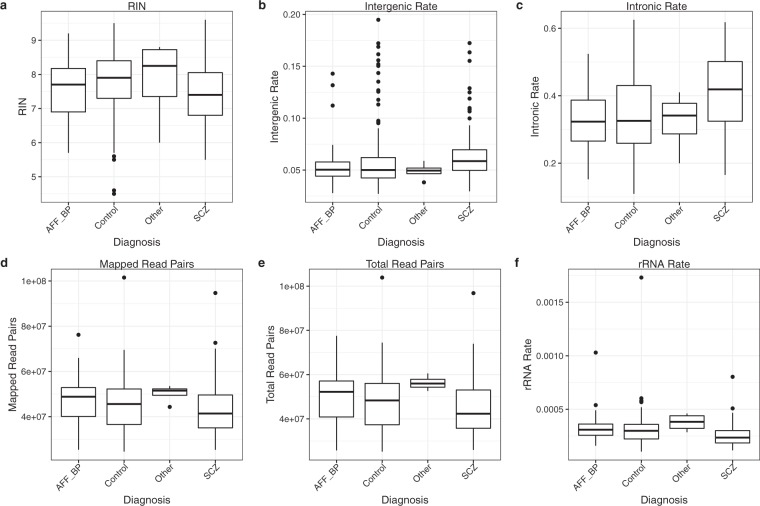
RNA-seq quality control metrics stratified by disease status. All samples from the 4 brain banks are shown.

**Fig. 2:**
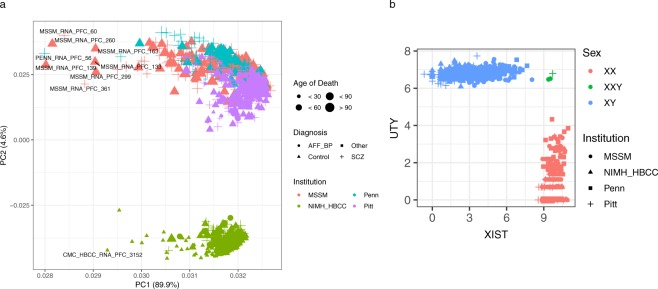
Integrated quality control of RNA-seq data. (**a**) Principal components analysis of log2 CPM values from RNA-seq across 4 brain banks. Brain bank, age of death, and diagnosis are indicated in the legend. (**b**) Plot of log2 CPM expression of UTY gene from chrY against XIST gene from chrX in order to validate reported sex.

All RNA-seq samples were integrated into a single analysis across the 4 brain banks in order to perform a joint quality control. Retained samples had acceptable values for RIN (mean 7.6, sd ±0.9), intergenic rate (mean 5.7%, sd ±1.9%), total read pairs (mean 4.6e + 7, sd ±1.1e + 7), intronic rate (mean 36.7%, sd ±11.2%), mapped read pairs (mean 4.42e + 7, sd ±9.8e + 6) and ribosomal RNA rate (mean 0.03%, sd ±0.01%) (Fig. 1 and Supplementary Fig. 2). Joint principal components analysis (PCA) of genes on autosomes identified outliers (Fig. 2a). As expected, PCA identified two distinct clusters separated along PC2 that correspond to technical differences in the RNA library preparation. Among other technical differences, samples from HBCC underwent a negative-strand library preparation, while the library preparation of samples from the remaining brain banks was not stranded. Joint downstream analysis must therefore include an RNA library preparation indicator variable as a covariate in order to account for this technical source of variation.

RNA-seq data was also used to confirm that the expression of genes on sex chromosomes is consistent with the reported sex. UTY and XIST were selected as represented genes, and samples show distinct clustering by reported sex after problematic samples were removed Fig. 2b).

### ATAC-seq quality control

Confirmation of the identity of samples and estimation of their contamination was performed by VerifyBamID (v1.1.3)^48^ based on the comparison of allele heterogeneity.

To evaluate the overall quality of samples, the peaks of open chromatin regions (OCRs) were called using MACS (v1.4.2)^49^. For the purpose of quality control analysis, the consensus peakset was created from the individual peaks that were subsequently merged across all samples, retaining only peaks found in at least 2 samples. Subsequently, we counted how many reads for each sample overlapped each consensus peak using featureCounts^27^ in the Rsubread package^50^. Finally, we counted fragments defined from paired-end reads that overlapped with the final consensus set of peaks.

The sex of the samples was assessed using three metrics: (1) the heterozygosity rate of chromosome X genotype calls outside the pseudoautosomal regions. For this, we discarded variants with MAF < 5%. In male samples, a high heterozygosity rate can indicate sample contamination, sex mismatch, or chromosomal abnormalities like Klinefelter’s syndrome. (2) The read counts of OCRs adjacent to FIRRE and XIST, which are only, or predominantly, accessible in females^51^. (3) Read counts in OCRs on chromosome Y outside the pseudoautosomal region.

**Fig. 3:**
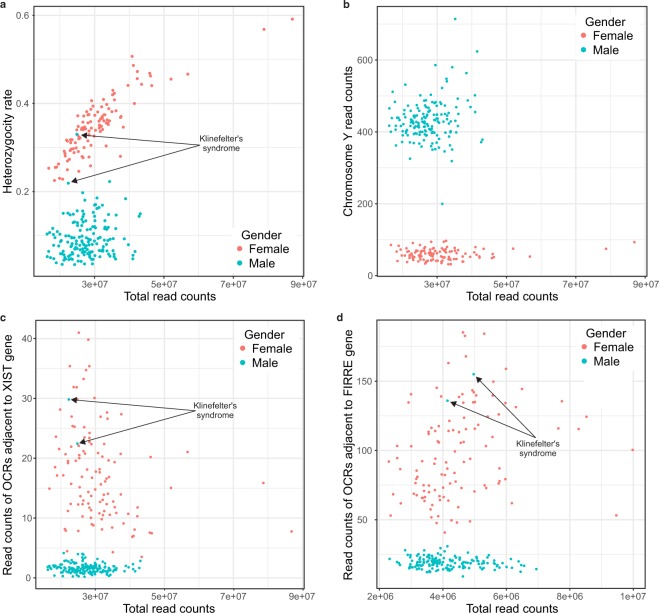
Sex check of ATAC-seq samples. (**a**) Heterozygosity rate of chromosome X genotype calls outside pseudoautosomal regions. (**b**) Read counts in OCRs on chromosome Y outside the pseudoautosomal region. (**c**,**d**) The read counts of OCRs adjacent to XIST and FIRRE genes.

**Fig. 4:**
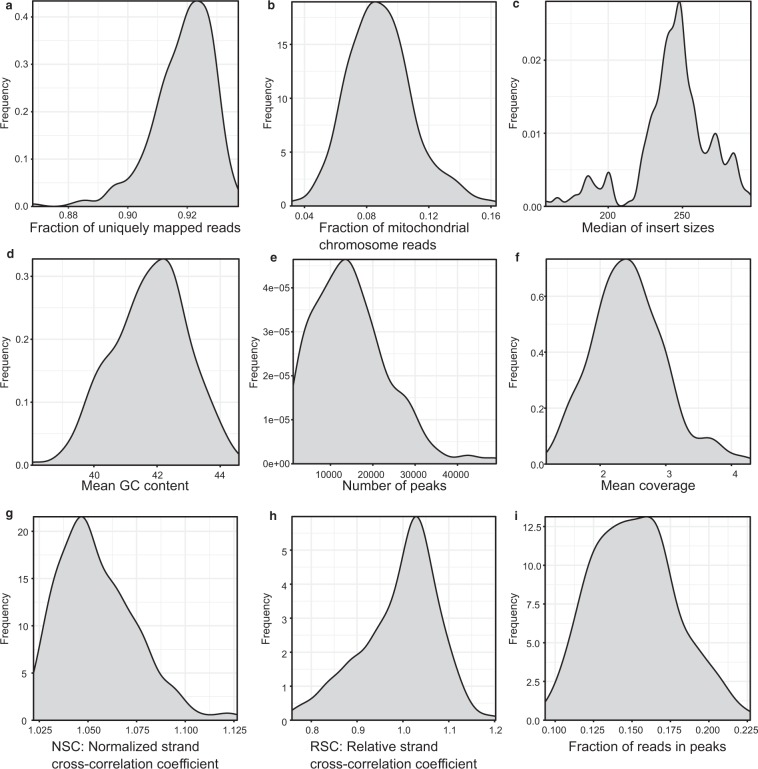
Quality control metrics for ATAC-seq samples. Histograms of (**a**) fraction of uniquely mapped reads (mean 0.919, sd ±0.010), (**b**) fraction of mitochondrial chromosome reads (mean 0.089, sd ±0.022), (**c**) mean insert sizes of pair-end reads (mean 288, sd ±29), (**d**) mean GC content (mean 0.418, sd ±0.012), (**e**) number of called peaks (mean 14,810, sd ±8,979), (**f**) mean coverage (mean 2.473, sd ±0.642), (**g**) normalized strand cross-correlation coefficient (mean 1.054, sd ±0.020), (**h**) relative strand correlation coefficient (mean 0.991, sd ±0.084)) and (**i**) fraction of fragments in peaks (FRiP) (mean 0.151, sd ±0.027).

**Fig. 5:**
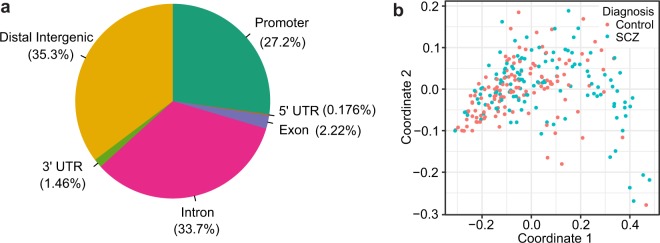
Summary of ATAC-seq data. (**a**) Genomic annotation of consensus OCRs (OCRs within 3 kb of a transcription start site were considered as promoter OCRs). (**b**) Clustering of the individual samples (n = 269) by chromatin accessibility in consensus OCRs using multidimensional scaling.

On average, we obtained 28.8 million uniquely mapped reads per sample after removing duplicate reads (mean 42%, sd ±9.5%) and those aligned to the mitochondrial genome (mean 8.9%, sd ±2.2%). Analyses included autosomes and sex chromosomes unless stated otherwise. The comparison of fractions of fragment in peaks (FRiP) was used to mark the replicates of lower quality for nine individuals that have more than one sample present in the dataset. Since we requested only one sample per individual, those nine replicates with lower FRiP were excluded. Using the sex check pipeline, two individuals were found to be genetically females though initially reported as males in the sample description (Fig. 3). However, further inspection revealed that they were diagnosed with Klinefelter’s syndrome that biases this observation. No other sex abnormalities or mismatches were observed. For nine individuals, VerifyBamID detected an improper matching between ATAC-seq and Illumina genotyping (see below). Therefore, these samples were excluded together with additional nineteen samples that were identified as possibly contaminated, leaving a final total of 269 samples (Fig. 4). Using this dataset, we generated a set of 272,424 peaks accounting for 4.96% of the genome (Fig. 5a). Finally, we quantified read counts of all the individual non-merged samples within these peaks and used these counts for MDS clustering (Fig. 5b).

### Genotype data quality control

**Fig. 6:**
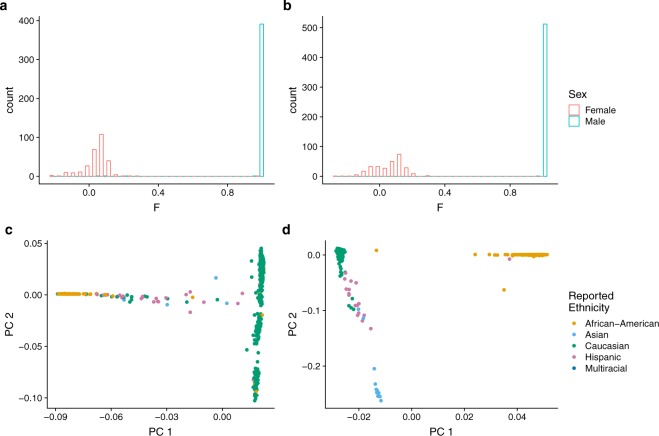
Quality control of genotype data. Genotype QC for sex (**a**,**b**) and ancestry inference (**c**,**d**) for MSSM-Penn-Pitt (**a**,**c**) and HBCC (**b**,**d**). (**a**,**b**) *F* statistic from plink’s *check-sex* function, plotted by reported sex. Following data QC there is 100% concordance between reported sex and inferred sex based on *F* statistic for both MSSM-Penn-Pitt (**a**) and HBCC (**b**). (**c**,**d**) The first two principal components (PC) of genetic ancestry as inferred by GEMTOOLs. For both MSSM-Penn-Pitt (**c**) and HBCC (**d**) we see good concordance between reported ethnicity and genetic background clusters inferred by GEMTOOLs.

Genotype data was analyzed to confirm reported sex and reported ethnicity (Fig. 6). As expected, the homozygosity rate on the X chromosome separated male and female samples in both the MSSM-Penn-Pitt cohort (Fig. 6a) and the HBCC cohort (Fig. 6b). Genetic ancestry was inferred using GEMTools^52^ on autosomes and showed good concordance with reported ethnicity.

### Variant concordance analysis across assays

**Fig. 7:**
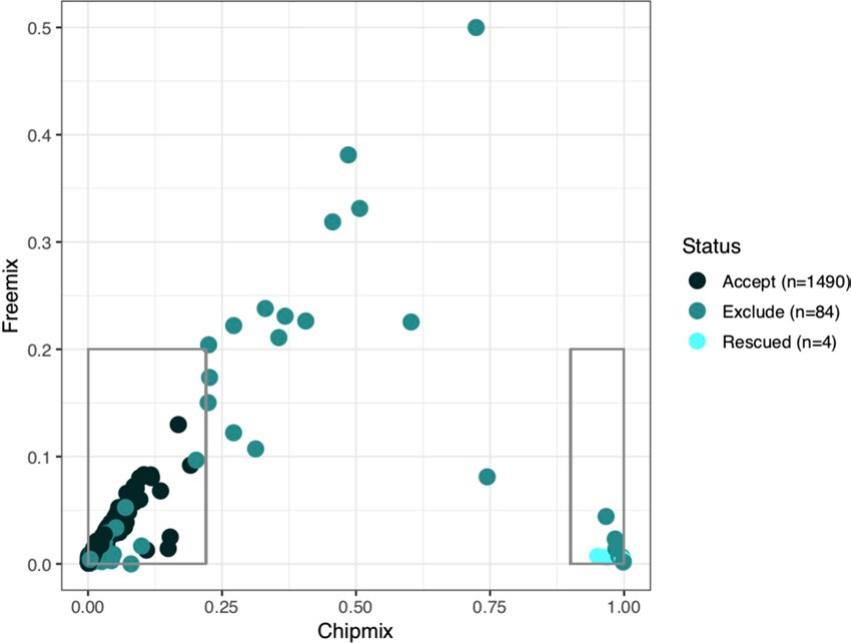
Assessing sample concordance using genetic variants. Estimating contamination using Chipmix (x-axis) and Freemix (y-axis) output from VerifyBamID, on RNA-seq and genotyping data for HBCC and MSSM-Penn-Pitt cohorts. Each point is an RNA-seq sample and is colored according to whether the sample was accepted, excluded or rescued. Box in lower left-hand corner indicates criteria for a sample to be accepted if samples match the expected individual. Box in lower right indicates samples that were rescued by re-labeling to the proper individual. We note that this figure included samples there were excluded because of other filters.

Integrated quality control across assays by evaluating concordance of genetic variants between SNP genotyping, RNA-seq and ATAC-seq. A simple approach would be to directly call genetic variants from RNA-seq and ATAC-seq and compare across assays. Since variant calling from functional genomics assays is technically challenging and has a high error rate, we used a statistical method to compare a BAM file from RNA-seq and ATAC-seq to the SNP genotyping data and evaluate the read support for each sample using only autosomes. BAM files using reads aligned to GRCh38 were compared to genotype data originally processed on GRCh37, but subsequently lifted overt to GRCh38. VerifyBamID^48^ was used to match RNA-seq data to SNP genotyping. Using the flags --best--ignoreRG--verbose, we identified each RNA-seq sample’s best genotyping match, and removed potentially contaminated samples, as judged by the chipmix and freemix estimates. If a BAM file matched to the expected individual in the SNP genotype data, the sample was accepted if its chipmix and freemix parameters were both below 0.2 (Fig. 7). Higher values for chipmix and freemix can indicate contamination, so samples exceeding this cutoff were excluded. Alternatively, If a BAM file did *not* match to the expected individual in the SNP genotype data, the sample was excluded except in 4 cases were the sample could be rescued by re-labeling it to the proper individual. Bcftools gtcheck was used to compare all whole genome sequencing and genotyping data. These data were subsetted to include only dbSNP non-AT/GC SNPs with MAF >0.25.

### Monkey RNA-seq quality control

Data provided passed the following QC filters: samples were required to have had a minimum of 50 million total reads and less than 5% rRNA alignment.

Enrichment and concordance of drug-related signatures from Fromer, *et al*.^9^ in Rhesus macaque treated with clozapine (N = 9) or haloperidol (N = 17), compared to untreated monkeys (N = 8) (Table 3). For the drugs tested, the number of human ortholog genes affected is listed, along with nominal p-value for enrichment of the gene signature in CMC differential expression data. Number of genes with concordant direction of effect between the gene signature in Fromer, *et al*.^9^, along with one-sided enrichment and depletion concordance p-values.

**Table 3:** Differential expression of RNA-seq data from macaque drug response trail.

Drug	Samples (drug/placebo)	Genes (n)	Enrichment (p-value)	Directional Concordance		
				n	Enrichment	Depletion
Clozapine	8/9	31	0.32	22	0.0125	0.9875
Haloperidol	8/17	237	1 × 10^−9^	35	>0.9999	<0.0001

## Usage Notes

### Use case 1: Differential expression analysis

Dysregulation of gene expression is of primary interest in understanding the molecular mechanisms of schizophrenia biology^9^. This CommonMind Consortium resource has RNA-seq data from postmortem brains from 351 schizophrenia cases and 500 controls. Researchers can perform differential expression analysis to identify genes whose expression differs significantly between cases and controls^53^. The 4 cohorts can be used to evaluate how well signals from one cohort replicate in an independent cohort. A meta-analysis can be performed by analyzing each cohort separately combining the results of the statistical test, or a mega-analysis can be performed by combining all cohorts into a single analysis.

### Use case 2: Differential chromatin accessibility

The role of epigenetic variation and in particular the variation in chromatin accessibility between schizophrenia cases and controls is of particular interest for its role in mediating downstream gene expression^21^. This resource includes ATAC-seq data from 126 schizophrenia cases and 127 controls. Researchers can perform statistical analysis of differential chromatin accessibility between cases and controls. These results can then be compared to the differential analysis of gene expression to examine the shared and unique components of these assays. Moreover, the chromatin accessibility data can be directly integrated with gene expression data where both assays were performed on the same samples.

### Use case 3: eQTL/caQTL analysis

Variants associated with schizophrenia are thought to mediate disease risk by regulating chromatin accessibility and then gene expression. By integrating genome-wide SNP genotyping data with RNA-seq from 981 individuals, researchers can identify eQTL’s. By integrating SNP genotyping data with ATAC-seq data, researchers can identify chromatin accessibility QTL’s (caQTL’s). These eQTL/caQTL results can then be integrated with summary statistics from genome-wide association studies (GWAS) of schizophrenia or other neuropsychiatric diseases to identify genes, or ATAC-seq peaks than mediate disease risk^54–56^.

### Use case 4: Coordinated regulation of epigenetics and gene expression

Integrated analysis of caQTLs, eQTLs, and GWAS summary statistics can trace the impact of risk variants from their downstream effect on a specific open chromatin region to the effect on gene expression and finally to disease risk^57^. This integrated framework can be used to understand epigenetic regulation of gene expression and further prioritize disease genes.

## Supplementary Information

Supplementary Information

## Data Availability

Source code, parameters for standard tools, and genome reference information is accessible via a central repository on Synapse at (http://CommonMind.org).
