# Supplementary material for: CommonMind Consortium provides transcriptomic and epigenomic data for Schizophrenia and Bipolar Disorder
Source: Sci Data. 2019 Sep 24;6:180. doi: 10.1038/s41597-019-0183-6 (PMC6760149; doi:10.1038/s41597-019-0183-6)
Supplement: Supplementary file 1 — Supplementary Information [file 41597_2019_183_MOESM1_ESM.pdf]

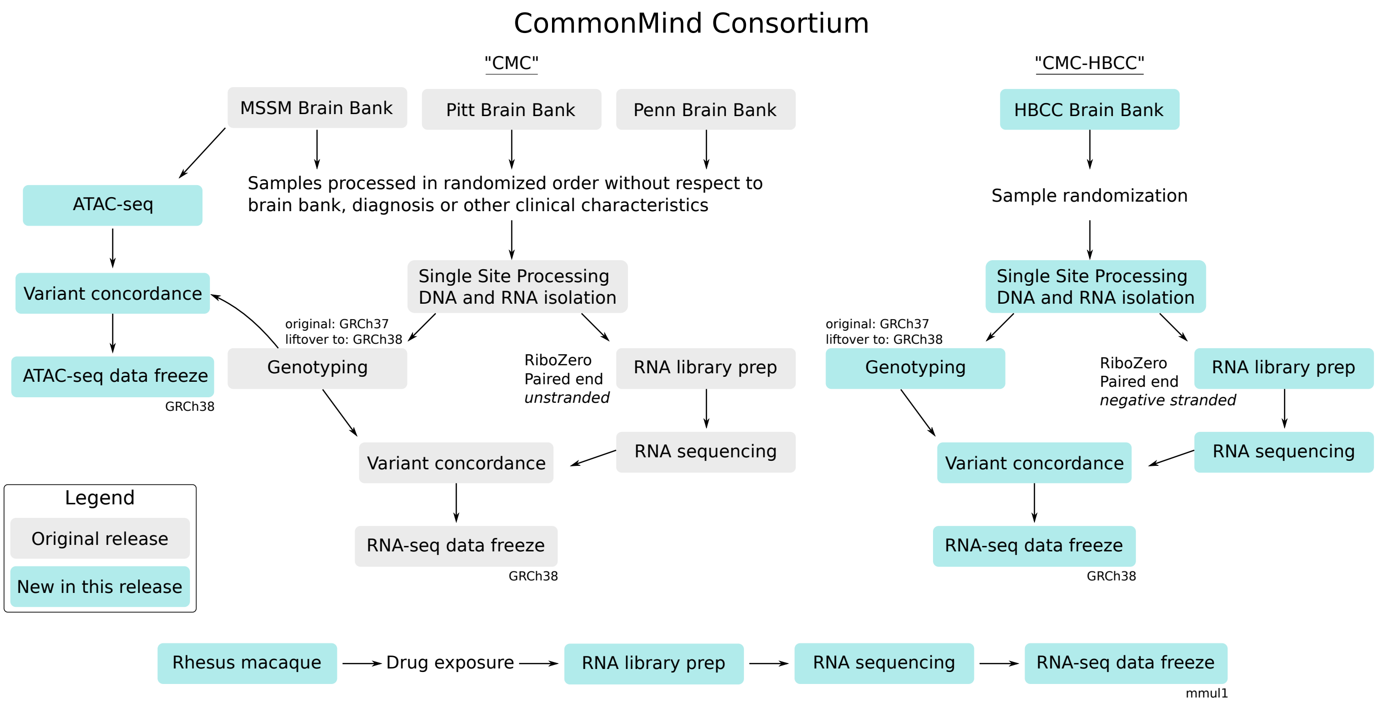

Supplementary Figure 1: Experimental workflow for four brain banks. Steps included in original release are shown in grey, steps new to the current release are shown in teal.

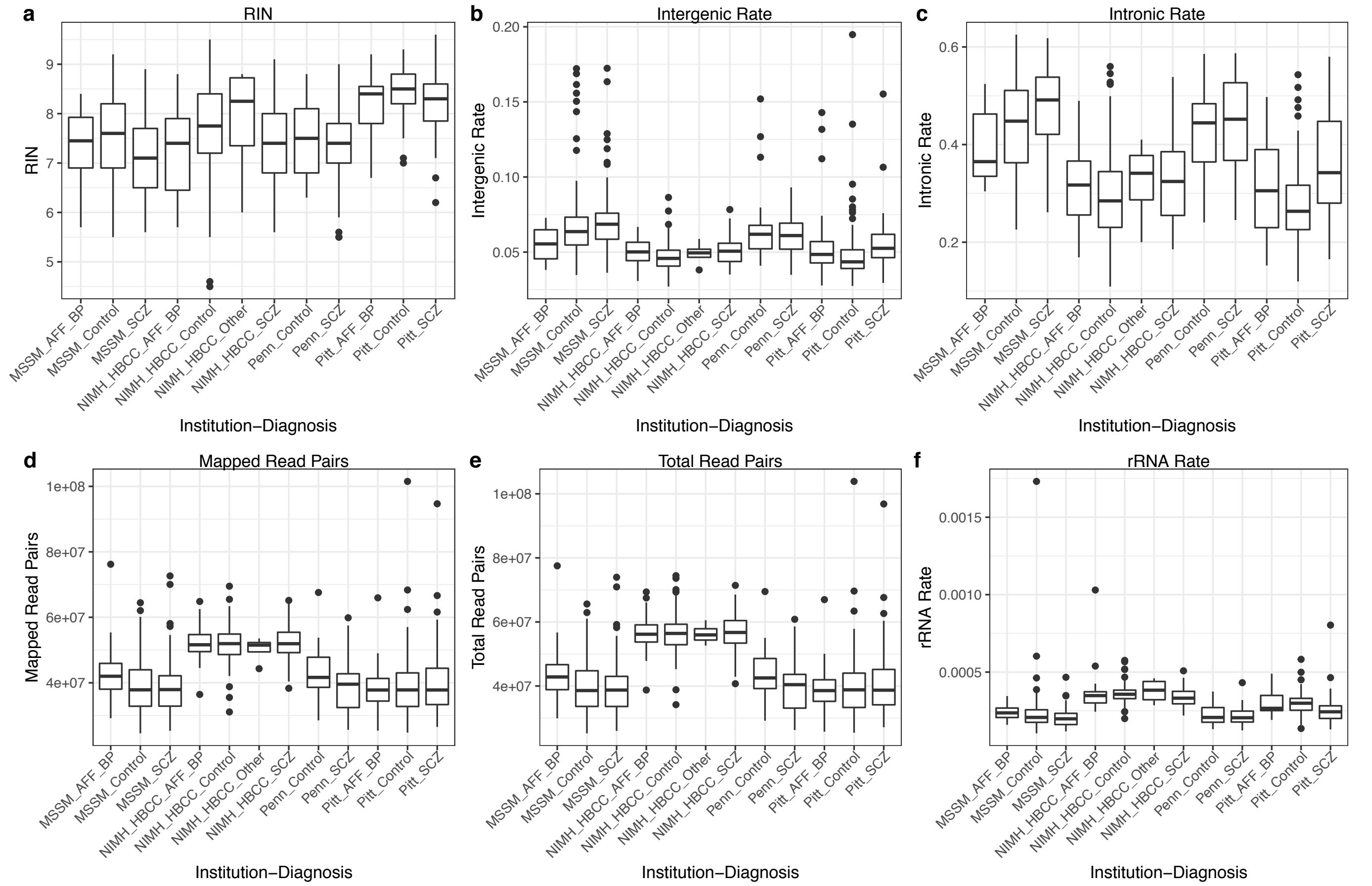

Supplementary Figure 2: RNA-seq quality control metrics stratified by brain bank and disease status. All samples from the 4 brain banks are shown.
